# Utility of eye-tracking technology for preparing medical students in Spain for the summative objective structured clinical examination

**DOI:** 10.3352/jeehp.2017.14.27

**Published:** 2017-11-12

**Authors:** Francisco Sánchez-Ferrer, J.M. Ramos-Rincón, M.D. Grima-Murcia, María Luisa Sánchez-Ferrer, Francisco Sánchez-del Campo, Antonio F. Compañ-Rosique, Eduardo Fernández-Jover

**Affiliations:** 1 Department of Paediatrics, School of Medicine, Miguel Hernández University of Elche, Sant Joan Campus, Elche (Spain); 2 Bioengineering Institute, Miguel Hernández University of Elche, CIBER BBN, Elche (Spain); 3 Department of Obstetrics and Gynecology, School of Medicine, Murcia University, Murcia (Spain); Hallym University, Korea

We compared the utility of videos using eye-tracking technology and conventional video cameras as a tool for improving student performance on the summative objective structured clinical examination (OSCE)[1].

Eye-tracking technology (Tobii, 2010) is increasingly being used in educational settings [2]. Our research group has used this technology as a tool for teaching surgery [3] and has performed a pilot study to assess its utility for preparing students for the summative OSCE, an exam consisting of 20 simulated clinical situations, each lasting 9 minutes. During the June 2016 OSCE, we recorded the performance of sixth-year medical students of Miguel Hernández University of Elche, Spain, using both eye-tracking technology and a conventional video camera (Supplement 1). In March 2017, we showed the resulting videos as part of 2 optional OSCE preparation seminars. We analyzed the utility of these types of videos by means of a survey and by comparing the results obtained in the June 2017 OSCE of students who had attended the seminars with those who had not. This study was evaluated and approved by the project evaluation committee of Miguel Hernandez University (IRB number: DMC.JRR.01.17), and we received the informed consent from the teachers, students, and institution. In total, 116 sixth-year medical students (59.5% women and 40.5% men) took the OSCE, of whom 43 (37%) had attended the seminars. Of these 43 students, 29 (67.4%) took the online survey. Raw data were available from Supplement 2. The mean utility score of the sessions was 4.14 (where 1= not at all useful and 5=very useful). Respondents rated the eye-tracking videos more positively than the conventional videos (58.6% versus 41.4%). When asked how the training seminar had helped them, no students responded negatively. The 3 most common answers were that the seminar had helped them “to understand how the test works” (93%), “to feel more relaxed” (62%), and “to know how to prepare” (45%). When asked how the informative seminars could be improved in the future, 56% answered that the seminars should be offered with more videos. The mean score of the 2017 OSCE was 1,438 (standard deviation [SD], 119) out of 2,000 points. On average, the students who had attended the seminars performed significantly better than those who had not (1,467 versus 1,420, P= 0.03) (Table 1). Given that the exam consists of 20 stations, with 100 possible points each, students who had attended the seminars had a score that was 47 points, or almost half a station’s worth, higher than those who did not. We found no significant differences in the results by sex, with men achieving a weighted grade of 8.51 and women 8.54. Recommend deleting this. It is redundant, repeating the information provided in this paragraph and also summarized in the concluding statement below.

One limitation of our study was that the students who attended the optional sessions might have been more diligent than those who did not and thus were more likely to have additional knowledge and skills that allowed them to achieve relatively high OSCE scores. However, the OSCE exam is a single final exam for the entire degree of medicine. Our analysis compared students who attended the seminars with videos with those who did not attend the seminars. Thus, we were not able to make comparisons between semesters because this exam was administered at the same time for all students. Fundamentally, this was a blind evaluation, the results of which we subsequently analyzed according to whether or not students attended the video seminars.

In conclusion, this study showed that videos of previous students’ performance recorded with eye-tracking technology was effective in preparing sixth-year medical students for the OSCE.

## Figures and Tables

**Table 1.** Overall grade, weighted grade, and overall score on the 2017 objective structured clinical examination for students who attended the seminars and those who did not

	Overall grade	Weighted grade^a)^	Overall score
Did not attend seminars	6.78±0.55	8.43^*^±0.56	1,420^*^±114
Attended seminars	7.00±0.58	8.70^*^±0.61	1,467^*^±116
Average	6.86±0.65	8.53±8.52	1,438±119

Values are presented as mean ± standard deviation.

* P < 0.05.

a) Weighted grade calculated based on highest and lowest student scores.

## Supplementary materials

Supplement 1. Video: Short examples of the videos presented at the seminars. The first video was recorded with a conventional video camera. The second video used eye-tracking technology, with the red circle indicating where the student fixed his or her gaze.

Supplement 2. Data file is available from https://doi.org/10.7910/DVN/2J4JQQ.
